# Nanostructured Silver Coating as a Stationary Phase for Capillary Gas Chromatography

**DOI:** 10.3390/molecules24244491

**Published:** 2019-12-08

**Authors:** Qiong Jiang, Peng Xu, Juanjuan Feng, Min Sun

**Affiliations:** 1 College of Plant Protection, Gansu Agricultural University/Biocontrol Engineering Laboratory of Crop Diseases and Pests of Gansu Province, Lanzhou 730070, China; xupeng@gsau.edu.cn; 2 Key Laboratory of Interfacial Reaction & Sensing Analysis in Universities of Shandong, School of Chemistry and Chemical Engineering, University of Jinan, Jinan 250022, China; chm_fengjuanjuan@ujn.edu.cn

**Keywords:** silver coating, nanomaterial, capillary column, gas chromatography, organic compounds

## Abstract

A capillary column coated with nanostructured silver coating was fabricated for gas chromatography. The nanostructured silver coating, about 80–120 nm in thickness, was prepared as the stationary phase via silver mirror reaction, and was characterized by SEM and EDS. The column was evaluated using different types of model analytes, including *n*-alkanes, *n*-alcohols, benzenes, and Grob mixture. A baseline separation of ten *n*-alkanes on the silver column (15 m × 0.20 mm i.d.) was achieved within 3.5 min through the main hydrophobic mechanism. A mixture of six *n*-alcohols, or another mixture containing three butanol isomers and two octanol isomers, was separated well on the column. The column separated some benzenes containing benzene, toluene, ethylbenzene, *p*-xylene, *o*-xylene, styrene, benzaldehyde, and benzyl alcohol. A Grob mixture containing seven analytes was also separated successfully. Based on a multiple retention mechanism such as hydrophobic, dipole-dipole, and dipole-induced dipole interactions, the silver column achieved a good separation of twelve different types of compounds within 2.5 min. The column presented satisfactory separation repeatability with relative standard deviation of retention time between 0.073% and 0.591%. The results indicate that the silver column is promising for gas chromatographic separation.

## 1. Introduction

Nanoparticles have attracted great attention due to their unique properties and potential in separation science. Various nanoparticles were applied for solid-phase extraction [1,2,3], solid-phase microextraction [4,5,6,7], removal of pollutants [8,9], and chromatographic stationary phase [10,11]. Carbon nanomaterials like carbon nanotubes (CNTs) and graphene oxide (GO) were used as the stationary phases for gas chromatography (GC), which exhibited excellent separation performance for various organic compounds. Based on a large number of active sites and the ability to provide π–π stacking [12], GC columns with CNTs could separate volatile aromatic or unsaturated organic compounds, such as polycyclic aromatic hydrocarbons, *n*-alkanes, and alcohols [13,14,15,16]. Using GO as the stationary phase separated a wide range of organic mixtures, including alcohols and aromatic compounds, which provided a high surface area to increase the phase ratio and rich functional groups for the formation of hydrophobic, hydrogen bonding, and π–π stacking interactions [17]. Graphitic carbon nitride-coated GC columns exhibited high-resolution capabilities for aromatic and aliphatic isomers such as methylnaphthalenes and dimethyl-naphthalenes, phenanthrene and anthracene, as well as alkane isomers [18], by advantage of unique retention mechanisms, including π–π stacking, hydrogen bonding, halogen bonding, and dispersion interactions. Gold nanoparticles modified with organic groups were also explored as the GC stationary phase [19,20,21,22]. A square capillary column coated with dodecanethiol monolayer-protected gold nanoparticles quickly separated a seven-component mixture (methyl ethyl ketone, benzene, octane, chlorobenzene, anisole, 3-octanone, and decane) in only 2 s [19,20,21]. The octadecylamine-capped gold nanoparticles were directly used to modify the capillary wall for GC, and the hydrophobic coating separated various types of analytes (benzene, 1-butanol, 1-pentanol, chlorobenzene, anisole) [22].

Silver nanoparticles have unique characteristics like good antibacterial activity [23] and adsorption performance for some organics [24]. Micro-structured silver coating or surface exhibited satisfactory extraction performance for polycyclic aromatic hydrocarbons and phthalate esters [25,26]. In order to explore the chromatographic performance of silver nanomaterials, a capillary column coated with a nanostructured silver coating was fabricated for GC in this work. The column was evaluated with different types of organic compounds, such as alkanes, bromoalkanes, alcohols, benzenes, and the Grob mixture.

## 2. Results and Discussion

### 2.1. Characterization of the Silver Capillary Column

**Figure 1:**
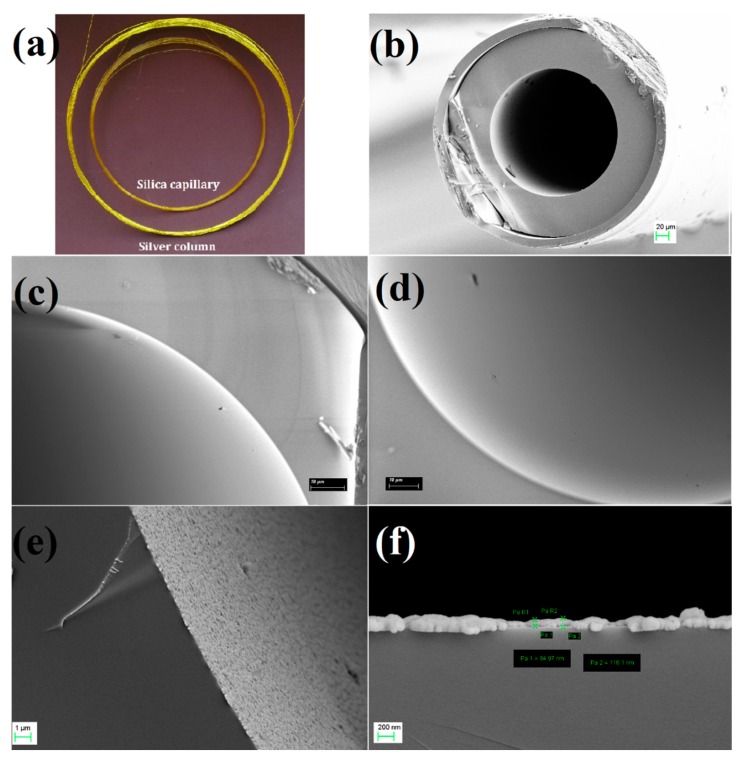
Photo of silver column (**a**) and SEM images (**b**–**f**), including the silver column (**b**), the inner surface of silica capillary (**c**), the inner surface of silica capillary modified with 3-mercaptopropyltrimethoxysilane (**d**), and the inner surface of silica capillary modified with silver coating (**e**, **f**).

After the modification of silica capillary with a silver coating, the silver capillary column shows a silver-white color in Figure 1a. As can be seen from Figure 1c, the inner surface of bare silica capillary is smooth, and no obvious change is observed after the modification by 3-mercaptopropyltrimethoxysilane in Figure 1d. However, the inner surface is densely covered by a rough coating through the functionalization of silver coating in Figure 1e. According to the EDS spectrum of the inner surface (Figure S1 in Supplementary material), the coating is confirmed as a silver coating. As shown in Figure 1f, the silver coating is very uniform with a thickness of about 80–120 nm. It was calculated as about 20 mg according to the concentration of [Ag(NH_3_)_2_]^+^ and the volume of capillary column. The silver coating is rough, which is favorable to chromatographic retention due to increasing active sites and contact area with the analyte.

### 2.2. Investigation of the Flow Rate

**Figure 2:**
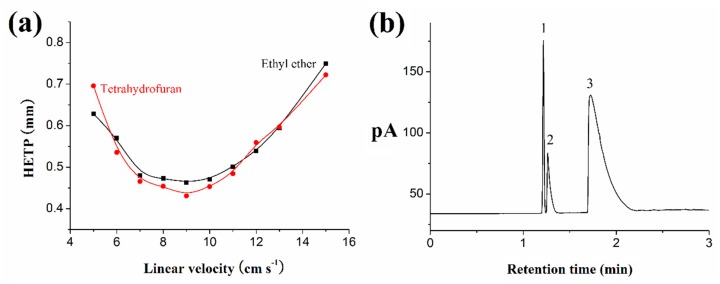
The Van Deemter plot for the silver column with ethyl ether and tetrahydrofuran at 55 °C (**a**), and the chromatogram of the polarity characterization (**b**). (**b**) Conditions: the carrier gas held at 0.3 mL min^−1^ for 1.2 min and up to 4 mL min^−1^ at a rate of 5 mL min^−1^; the injection volume was 0.01 μL; the injection split was 100:1. Peaks: cyclohexane (1), benzene (2), and pyridine (3).

It is known that the chromatographic behavior of the column is dependent on the flow rate of the carrier gas. As shown in Figure 2a, the Van Deemter plot of the silver column is investigated with ethyl ether and tetrahydrofuran at 55 °C. The highest column efficiency occurs under a linear velocity of around 9 cm s^−1^. The minimum height equivalent theoretical plate (HETP) is 0.46 mm for ethyl ether and 0.43 mm for tetrahydrofuran, which correspond to 2174 and 2326 plates m^−1^, respectively. It also indicated that the column efficiency was only slightly affected, even when a higher carrier gas flow rate was used.

### 2.3. Characterization of the Polarity of Silver Column

Three compounds containing cyclohexane (b.p. 80.7 °C), benzene (b.p. 80.1 °C), and pyridine were used to evaluate the polarity of the silver column. Cyclohexane and benzene are nonpolar compounds, but benzene is easily polarized. Pyridine is polar. If the silver column is a nonpolar column, the retention time of cyclohexane should be larger than that of benzene. If it is a polar column, pyridine should be more retained than benzene. Figure 2b shows that benzene is more retained than cyclohexane, indicating that the silver column is polar. Pyridine is more retained than benzene, which also further illustrates the polarity of the silver column.

### 2.4. Chromatographic Separation of the Silver Column

#### 2.4.1. Separation of Alkanes

**Figure 3:**
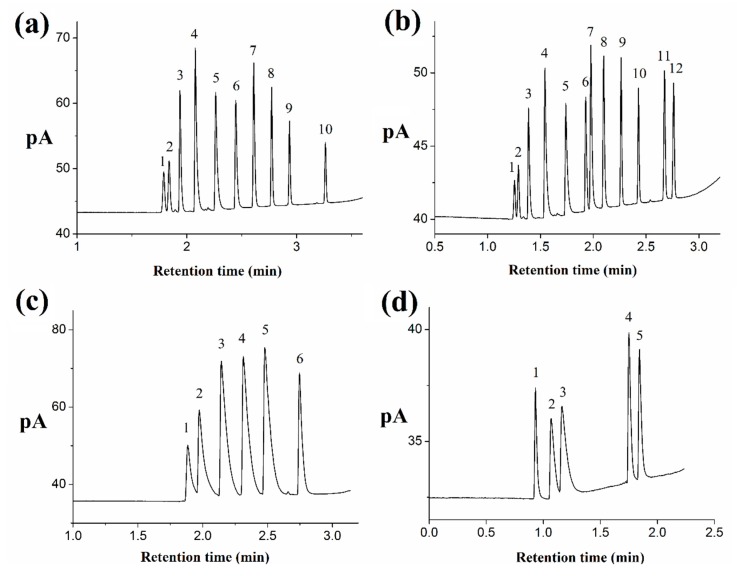
The chromatographic separation of ten *n*-alkanes (**a**), *n*-alkanes and 1-bromoalkanes (**b**), six *n*-alcohols (**c**), and the butanol isomers (**d**). (**a**) *Conditions*: held at 30 °C for 1.8 min and up to 140 °C for 15 min at a rate of 50 °C min^−1^; the flow rate of the carrier gas held at 0.2 mL min^−1^ (10 cm s^−1^) for 1.8 min and up to 3 mL min^−1^ at a rate of 2 mL min^−1^; the injection volume was 0.02 μL; the injection split was 100:1. Chromatographic peaks: *n*-hexane (1), *n*-heptane (2), *n*-octane (3), *n*-nonane (4), *n*-decane (5), *n*-undecane (6), *n*-dodecane (7), *n*-tridecane (8), *n*-tetradecane (9), and *n*-hexadecane (10). (**b**) *Conditions*: held at 30 °C for 1.3 min and up to 140 °C for 15 min at a rate of 70 °C min^−1^; the flow rate of the carrier gas held at 0.3 mL min^−1^ for 1.3 min and up to 3 mL min^−1^ at a rate of 2 mL min^−1^; the injection volume was 0.02 μL; the injection split was 100:1. Chromatographic peaks: *n*-hexane (1), *n*-heptane (2), *n*-octane (3), *n*-nonane (4), *n*-decane (5), *n*-undecane (6), 1-bromooctane (7), *n*-dodecane (8), *n*-tridecane (9), *n*-tetradecane (10), *n*-hexadecane (11), and 1-bromododecane (12). (**c**) *Conditions*: held at 28 °C for 2 min and up to 100 °C at a rate of 80 °C min^−1^; the flow rate of carrier gas held at 0.2 mL min^−1^ for 2 min and up to 3 mL min^−1^ at a rate of 3 mL min^−1^; the injection volume was 0.02 μL; the injection split was 100:1. Chromatographic peaks: methanol (1), *n*-propanol (2), *n*-butanol (3), *n*-pentanol (4), *n*-hexyl alcohol (5), and *n*-octanol (6). (**d**) *Conditions*: held at 25 °C for 1 min and up to 100 °C at a rate of 60 °C min^−1^; the flow rate of carrier gas from 0.46 mL min^−1^ (20 cm s^−1^) up to 3 mL min^−1^ at a rate of 3 mL min^−1^; the injection volume was 0.02 μL; the injection split was 100:1. Chromatographic peaks: *tert*-butanol (1), isobutanol (2) *n*-butanol (3), ethylhexanol (4), and *n*-octanol (5).

The chromatographic separation of *n*-alkanes with a carbon number of about 6–14 and 16 was tested on the silver column. The chromatographic parameters were optimized to achieve a baseline separation for ten components. As shown in Figure 3a, the baseline separation of all *n*-alkanes was completed in only 3.5 min with the column temperature ramping from 30 °C (held 1.8 min) to 140 °C at a rate of 50 °C min^−1^ and the flow rate of the carrier gas ramping from 0.2 mL min^−1^ (held 1.8 min) to 3 mL min^−1^ at a rate of 2 mL min^−1^. The elution sequence follows an increasing order of boiling points and the carbon numbers of all the analytes. Silver coating with a good control over surface roughness can produce hydrophobic characteristic [27,28,29]. Considering the nonpolarity and hydrophobicity of *n*-alkanes, the chromatographic separation might be attributed to van der Waals force between the linear alkanes and the hydrophobic silver coating of the column. In addition, the retention interaction is closely related to the carbon number of *n*-alkanes. Several 1-bromoalkanes were also separated well on the silver column based on this chromatographic mechanism (Figure S2 in Supplementary material). Furthermore, a mixture containing ten *n*-alkanes and two 1-bromoalkanes was successfully separated in only 3.0 min (Figure 3b).

#### 2.4.2. Separation of Alcohols

Alcohols were also applied to investigate the silver column. Six *n*-alcohols are separated in Figure 3c, but their peaks are tailing. The elution sequence follows an increasing order of their boiling points (methanol 64.5 °C, *n*-propanol 97.1 °C, *n*-butanol 117.7 °C, *n*-pentanol 128 °C, *n*-hexyl alcohol 157.2 °C, and *n*-octanol 195 °C). Hydrophobic interaction may play a main role in the retention of alcohols on the column, the irreversible adsorption sites, and the polar property of silver stationary phase result in tailing peaks. The silver column can separate isomers of alcohols. Three butanol isomers (*tert*-butanol, isobutanol, and *n*-butanol) and two octanol isomers (ethylhexanol and *n*-octanol) are separated well in Figure 3d.

#### 2.4.3. Separation of Benzenes

**Figure 4:**
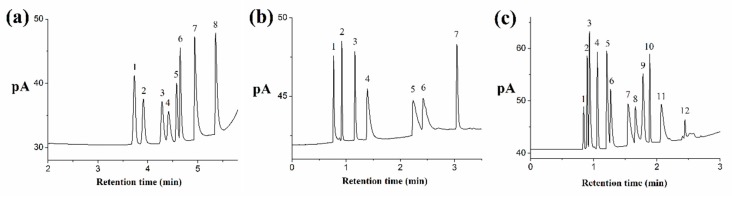
The chromatographic separation of benzenes (**a**), the Grob mixture (**b**), and the mixture of different types of compounds (**c**). (**a**) *Conditions*: held at 28 °C for 4.5 min and up to 140 °C at a rate of 80 °C min^−1^; the flow rate of carrier gas from 0.1 mL min^−1^ up to 4 mL min^−1^ at a rate of 4.5 mL min^−1^; the injection volume was 0.02 μL; the injection split was 100:1. Chromatographic peaks: benzene (1), toluene (2), ethylbenzene (3), *p*-xylene (4), *o*-xylene (5), styrene (6), benzaldehyde (7), and benzyl alcohol (8). (**b**) *Conditions*: held at 50 °C for 1.5 min and up to 100 °C at a rate of 60 °C min^−1^; the carrier gas was 0.5 mL min^−1^; the injection volume was 0.02 μL; the injection split was 100:1. Chromatographic peaks: methanol (1), *n*-decane (2), *n*-undecane (3), *n*-nonaldehyde (4), 2,6-dimethylaniline (5), 2,6-dimethylphenol (6), and methyl dodecanoate (7). (**c**) *Conditions*: held at 35 °C for 1 min and up to 140 °C at a rate of 60 °C min^−1^; the carrier gas held at 0.5 mL min^−1^ for 1 min and up to 3 mL min^−1^ at a rate of 3 mL min^−1^; the injection volume was 0.02 μL; the injection split was 100:1. Chromatographic peaks: *n*-hexane (1), *n*-heptane (2), 1-bromobutane (3), *n*-octane (4), *p*-xylene (5), *o*-xylene (6), cyclohexanone (7), benzaldehyde (8), 1-bromooctane (9), *n*-dodecane (10), benzyl alcohol (11), and 1-bromododecane (12).

As shown in Figure 4a, some benzenes containing benzene, toluene, ethylbenzene, *p*-xylene, *o*-xylene, styrene, benzaldehyde, and benzyl alcohol are successfully separated by the silver column. Except the resolutions of ethylbenzene/*p*-xylene and *o*-xylene/styrene, which are less than 2, the resolutions among other benzenes are larger than 2. Chromatographic separation of ethylbenzene and xylene isomers is challenging because of their coherent boiling points and dimensions. The silver column can efficiently separate these compounds. The resolutions of ethylbenzene/*p*-xylene and *p*-xylene/*o*-xylene are 1.22 and 2.01, respectively. Compared with ethylbenzene, styrene is better retained on the silver column due to its larger π-electron.

#### 2.4.4. Separation of the Grob Mixture

The Grob mixture is a test mixture for the comprehensive assessment of the overall chromatographic performance and activity of the GC column. Resolution, elution order, and peak shape of analytes are indicative of their retention behavior on the given column [30]. Figure 4b shows the chromatogram of a Grob mixture containing seven analytes (methanol, *n*-decane, *n*-undecane, *n*-nonaldehyde, 2,6-dimethylaniline, 2,6-dimethylphenol, and methyl dodecanoate). 2,6-Dimethylphenol presents stronger retention than 2,6-dimethylaniline, indicating the alkaline property of the silver column. Chromatographic peaks of *n*-nonaldehyde, 2,6-dimethylphenol, and 2,6-dimethylaniline are tailing due to the very polar property of the silver column. *n*-Decane, *n*-undecane, and methyl dodecanoate show symmetrical peaks with the best column efficiency of 72,673 plates m^−1^.

#### 2.4.5. Separation of Different Types of Compounds

A mixture of different types of compounds (*n*-alkanes, bromoalkanes, benzenes, ketones, and aldehydes) was applied to investigate the comprehensive chromatographic performance of the silver column. A sample composed of *n*-hexane, *n*-heptane, 1-bromobutane, *n*-octane, *p*-xylene, *o*-xylene, cyclohexanone, benzaldehyde, 1-bromooctane, *n*-dodecane, benzyl alcohol, and 1-bromododecane was successfully separated within 2.5 min in Figure 4c. The chemical structure, column efficiency and resolution of these compounds are shown in Table 1. This indicates that the silver column possesses a high separation ability for different types of compounds through mixed retention mechanisms such as hydrophobic, dipole–dipole, and dipole-induced dipole interactions.

#### 2.4.6. Repeatability of Chromatographic Separation

Separation repeatability of the GC column is very important for its application. The column was tested by a repeated injection of the *n*-alkanes mixture (ten times). The chromatograms are shown in Figure S3. The relative standard deviations of retention times for ten *n*-alkanes are in the range of 0.073%–0.591%. According to the results, the silver column exhibits a satisfactory chromatographic repeatability.

## 3. Materials and Methods

### 3.1. Materials and Reagents

3-Mercaptopropyltrimethoxysilane (98%) was obtained from Qufu Chenguang Fine Chemical Co. (Qufu, China) and was purified by vacuum distillation before use. Toluene was dried by refluxing with sodium for 24 h and was distilled before use. AgNO_3_ and glucose were of analytical-grade quality and were obtained from Sinopharm Chemical Reagent Co. (Shanghai, China). All the other chemicals were of analytical-grade quality. The silica capillary (0.20 mm, i.d.) was purchased from Yongnian Ruifeng Chromatographic Apparatus Co., Ltd. (Handan, Hebei, China).

### 3.2. Apparatus

An Agilent 7890A GC system (Agilent Technologies, Palo Alto, California, USA) equipped with a flame ionization detector (FID) and a split/splitless inlet was used. All chromatographic separations were performed under the following conditions: ultrapure nitrogen (>99.999%) as a carrier gas and make-up gas, injection port at 200 °C, split injection mode at a ratio of 100:1, and FID detector at 300 °C. A field-emission scanning electron microscope (SEM, SUPRATM55, Carl Zeiss, AG, Heidenheim, Germany) and an energy-dispersive X-ray spectrometer (EDS, Oxford INCA X-Act, High Wycombe, England) were used for the characterization.

### 3.3. Preparation of the Silver Column

A silica capillary (15 m × 0.20 mm i.d.) was filled with 0.1 mol L^−1^ NaOH solution under 65 °C for 1 h to expose the silanol groups on inner surface. Then, the capillary was successively rinsed with 0.1 mol L^−1^ HCl and water for 60 min and 30 min, respectively. Afterwards, the pretreated capillary was rinsed with methanol for 5 min and dried by nitrogen flow for the following process.

In order to strengthen the binding between the silver coating and the inner surface of the silica capillary, the capillary was modified by the mercapto groups before the functionalization with silver coating. Firstly, 0.5 mL of 3-mercaptopropyltrimethoxysilane was dissolved in 2.0 mL of toluene. Next, the above solution was injected into the pretreated silica capillary and stayed at 110 °C for 24 h, and then the capillary was successively rinsed with toluene, ethanol, water, and methanol.

The functionalization of the capillary was performed by a modified method in our previous report [25]. The capillary modified with mercapto groups was filled by a reaction solution containing 0.4 mol L^−1^ of [Ag(NH_3_)_2_]^+^ and 0.4 mol L^−1^ of glucose at room temperature for 3 h. After the modification, the capillary column presented a silvery color (Figure 1a). Then, the column with the silver coating was rinsed with water and methanol in turn. After being dried, the silver column was conditioned with 0.5 mL min^−1^ N_2_ from 50 °C to 150 °C at a rate of 5 °C min^−1^ and held at the final temperature for 3 h.

## 4. Conclusions

In this work, a GC capillary column with a nanostructured silver coating was developed by a silver mirror reaction. The silver coating, about 80–120 nm in thickness, worked as the stationary phase, which could interact with various organic compounds, including *n*-alkanes, *n*-alcohols, 1-bromoalkanes, and benzenes. The good chromatographic separation of these types of compounds could be obtained on the silver column, respectively. Furthermore, the silver column could separate twelve different types of compounds within 2.5 min, based on a multiple retention mechanism, such as hydrophobic, dipole-dipole, and dipole-induced dipole interactions. This demonstrates that the silver column is promising for gas chromatographic separation. Future work will focus on the modification of the silver column by functionalizing the silver coating for further chromatographic separation.

## Figures and Tables

**Table 1:** Twelve compounds separated by the silver column in Figure 4c.

Compounds	Boiling Point	Chemical Structure	Column Efficiency (Plates m^−^^1^)	Resolution
*n*-Hexane	68.74	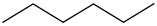	12013	----
*n*-Heptane	98.5	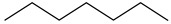	5657	1.26
*n*-Butyl bromide	101.6	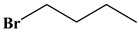	8864	1.09
*n*-Octane	125.8	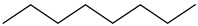	14847	3.31
*p*-Xylene	138.5	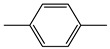	2847	2.58
*o*-Xylene	144.4	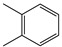	11131	0.43
Cyclohexanone	155.6	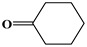	1103	2.80
Benzaldehyde	179	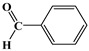	6493	0.77
1-Bromooctane	201	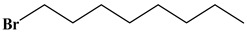	17142	1.34
*n*-dodecane	213	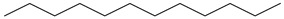	74679	2.63
Benzyl alcohol	205.4	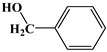	9754	3.30
1-Bromododecane	276	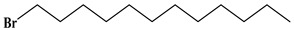	110382	6.57

## Supplementary Materials

The supplementary materials are available online https://www.mdpi.com/1420-3049/24/24/4491/s1.
