# Supplementary material for: Nanostructured Silver Coating as a Stationary Phase for Capillary Gas Chromatography
Source: Molecules. 2019 Dec 8;24(24):4491. doi: 10.3390/molecules24244491 (PMC6943660; doi:10.3390/molecules24244491)
Supplement: Supplementary file 1 [file molecules-24-04491-s001.pdf]

Supplementary material

Nanostructured silver coating as a stationary phase for capillary gas chromatography

Qiong Jiang [1,*], Peng Xu [1], Juanjuan Feng [2] and Min Sun [2,*]

[1] College of Plant Protection, Gansu Agricultural University/ Biocontrol Engineering Laboratory of Crop Diseases and Pests of Gansu Province, Lanzhou, Gansu Province 730070, China; menghuanrou@163.com (Q. Jiang); xupeng@gsau.edu.cn (P. Xu)

[2] Key Laboratory of Interfacial Reaction & Sensing Analysis in Universities of Shandong, School of Chemistry and Chemical Engineering, University of Jinan, Jinan 250022, PR China; chm_fengjuanjuan@ujn.edu.cn (J. Feng); chm_sunm@ujn.edu.cn (M. Sun)

* Correspondence: menghuanrou@163.com (Q. Jiang); Tel.: +86-931-7632260.

* Correspondence: chm_sunm@ujn.edu.cn (M. Sun); Tel.: +86-531-89736065.

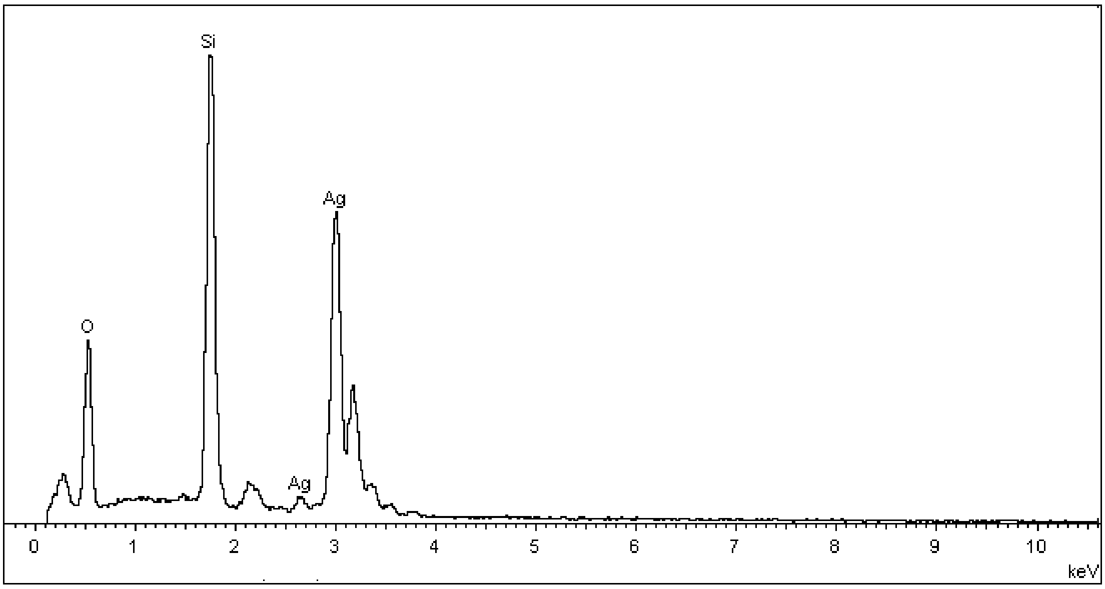

Fig. S1. The EDS spectrum of the inner surface of silver column.

Table S1 The percentage of the inner surface of silver column from EDS characterization.

elements	weight percentage (%)	atom percentage (%)
Ag	51.79	16.71
Si	23.06	28.58
O	25.15	54.71

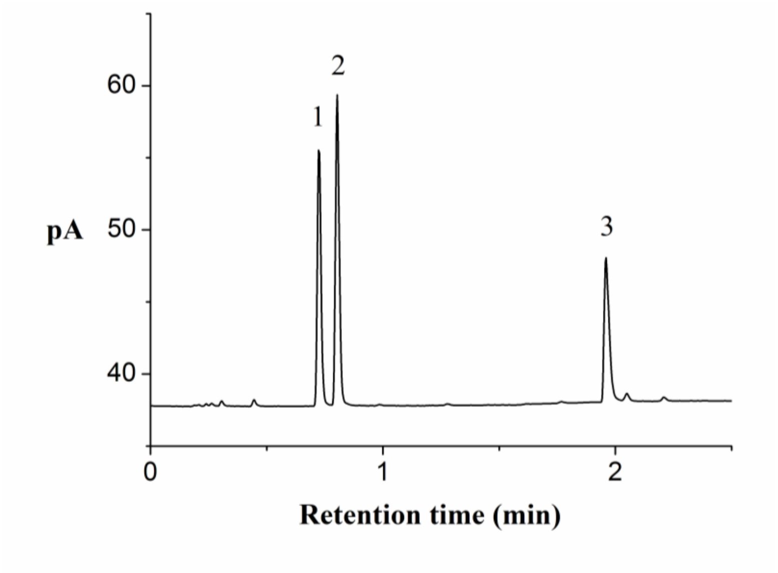

Fig. S2. The chromatogram of 1-bromoalkanes on the silver column. Chromatographic peaks: 1-bromobutane (1), 1-bromooctane (2) and 1-bromododecane (3). Conditions: column temperature at 80 °C; the flow rate of carrier gas held at 0.5 mL min^{-1} (22 cm s^{-1}) and up to 3 mL min^{-1} at a rate of 3 mL min^{-1}; the injection volume was 0.01 μL; the injection split was 50:1.

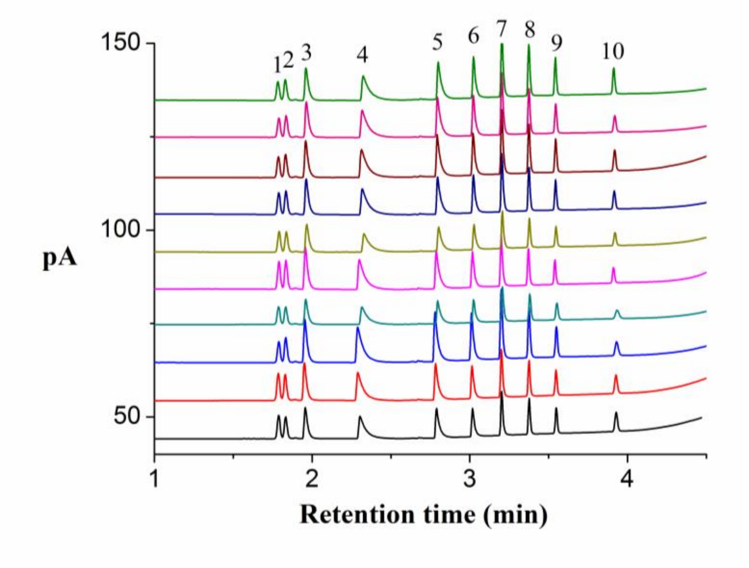

Fig. S3. Repeatability of chromatographic separation of *n*-alkanes on the silver column. Chromatographic peaks: *n*-hexane (1), *n*-heptane (2), *n*-octane (3), *n*-nonane (4), *n*-decane (5), *n*-undecane (6), *n*-dodecane (7), *n*-tridecane (8), *n*-tetradecane (9) and *n*-hexadecane (10). Conditions: column temperature held at 31 °C for 2.5 min and up to 140 °C for 15 min at a rate of 50 °C min^{-1}; the flow rate of carrier gas held at 0.3 mL min^{-1} (15 cm s^{-1}) for 2.5 min and up to 3 mL min^{-1} at a rate of 3 mL min^{-1}; the injection volume was 0.01 μL; the injection split was 100:1.
